# The future of Blue Carbon science

**DOI:** 10.1038/s41467-019-11693-w

**Published:** 2019-09-05

**Authors:** Peter I. Macreadie, Andrea Anton, John A. Raven, Nicola Beaumont, Rod M. Connolly, Daniel A. Friess, Jeffrey J. Kelleway, Hilary Kennedy, Tomohiro Kuwae, Paul S. Lavery, Catherine E. Lovelock, Dan A. Smale, Eugenia T. Apostolaki, Trisha B. Atwood, Jeff Baldock, Thomas S. Bianchi, Gail L. Chmura, Bradley D. Eyre, James W. Fourqurean, Jason M. Hall-Spencer, Mark Huxham, Iris E. Hendriks, Dorte Krause-Jensen, Dan Laffoley, Tiziana Luisetti, Núria Marbà, Pere Masque, Karen J. McGlathery, J. Patrick Megonigal, Daniel Murdiyarso, Bayden D. Russell, Rui Santos, Oscar Serrano, Brian R. Silliman, Kenta Watanabe, Carlos M. Duarte

**Affiliations:** 1 Deakin University, School of Life and Environmental Sciences, Center for Integrative Ecology, Geelong, VIC 3125 Australia; 2 King Abdullah University of Science and Technology, Red Sea Research Center and Computational Bioscience Research Center, Thuwal, Saudi Arabia; 3 Division of Plant Sciences, University of Dundee at the James Hutton Institute, Invergowrie, Dundee, DD2 5DQ UK; 4 Climate Change Cluster, University of Technology Sydney, Ultimo, NSW 2007 Australia; 5 School of Biological Science, University of Western Australia, 35 Stirling Highway, Crawley, WA 6009 Australia; 6 Plymouth Marine Laboratory, Prospect Place, Plymouth, PL1 3DH UK; 7 Australian Rivers Institute—Coast & Estuaries, School of Environment and Science, Griffith University, Gold Coast, QLD 4222 Australia; 8 Department of Geography, National University of Singapore, 1 Arts Link, Singapore, 117570 Singapore; 9 School of Earth, Atmospheric and Life Sciences, University of Wollongong, Wollongong, NSW 2522 Australia; 10 School of Ocean Sciences, Bangor University, Menai bridge, Bangor, LL59 5AB UK; 11 Coastal and Estuarine Environment Research Group, Port and Airport Research Institute, 3-1-1 Nagase, Yokosuka, 239-0826 Japan; 12 School of Science, Centre for Marine Ecosystems Research, Edith Cowan University, 270 Joondalup Drive, Joondalup, WA 6027 Australia; 13 School of Biological Sciences, The University of Queensland, St Lucia, QLD 4072 Australia; 14 Marine Biological Association of the United Kingdom, Citadel Hill, Plymouth, PL1 2PB UK; 15 Institute of Oceanography, Hellenic Centre for Marine Research, PO Box 2214, 71003 Heraklion, Crete Greece; 16 Department of Watershed Sciences and Ecology Center, Utah State University, Logan, UT 84322-5210 USA; 17 CSIRO Agriculture and Food, Private Mail Bag, Glen Osmond, SA 5064 Australia; 18 Department of Geological Sciences, University of Florida, Gainesville, FL 32611-2120 USA; 19 Department of Geography, McGill University, 805 Sherbrooke St W, Montreal, QC H3A 0B9 Canada; 20 Centre for Coastal Biogeochemistry, School of Environment, Science and Engineering, Southern Cross University, Lismore, NSW 2480 Australia; 21 Department of Biological Sciences and Center for Coastal Oceans Research, Florida International University, 11200 SW8th St, Miami, FL 33199 USA; 22 School of Biological and Marine Sciences, University of Plymouth, Plymouth, UK; 23 Shimoda Marine Research Center, University of Tsukuba, Tsukuba, Japan; 24 School of Applied Sciences, Edinburgh Napier University, Edinburgh, EH11 4BN UK; 25 Global Change Research Group, IMEDEA (CSIC-UIB), Institut Mediterrani d’Estudis Avançats, Miquel Marquès 21, Esporles, 07190 Spain; 26 Department of Bioscience, Aarhus University, Vejlsøvej 25, Silkeborg, 8600 Denmark; 27 Arctic Research Centre, Department of Bioscience, Aarhus University, Ny Munkegade 114, bldg. 1540, Århus C, 8000 Denmark; 28 World Commission on Protected Areas, IUCN, Gland, Switzerland; 29 Centre for Environment, Fisheries, and Aquaculture Science, Lowestoft, UK; 30 The Oceans Institute and Department of Physics, The University of Western Australia, 35 Stirling Highway, Crawley, WA Australia; 31 Departament de Física & Institut de Ciència i Tecnologia Ambientals, Universitat Autònoma de Barcelona, Bellaterra, 08193 Spain; 32 Department of Environmental Sciences, University of Virginia, Charlotttesville, VA 22903 USA; 33 Smithsonian Environmental Research Center, 647 Contees Wharf Road, Edgewater, MD 21037 USA; 34 Center for International Forestry Research (CIFOR), Jl. CIFOR, Situgede, Bogor, 16115 Indonesia; 35 Department of Geophysics and Meteorology, Bogor Agricultural University, Kampus Darmaga, Bogor, 16680 Indonesia; 36 Swire Institute of Marine Science, School of Biological Sciences, University of Hong Kong, Hong Kong SAR, China; 37 Center of Marine Sciences, CCMAR, University of Algarve, Faro, 8005-139 Portugal; 38 Nicholas School of the Environment, Duke University, 135 Duke Marine Lab Road, Beaufort, NC 28516 USA

**Keywords:** Microbiology, Plant sciences, Biogeochemistry

## Abstract

The term Blue Carbon (BC) was first coined a decade ago to describe the disproportionately large contribution of coastal vegetated ecosystems to global carbon sequestration. The role of BC in climate change mitigation and adaptation has now reached international prominence. To help prioritise future research, we assembled leading experts in the field to agree upon the top-ten pending questions in BC science. Understanding how climate change affects carbon accumulation in mature BC ecosystems and during their restoration was a high priority. Controversial questions included the role of carbonate and macroalgae in BC cycling, and the degree to which greenhouse gases are released following disturbance of BC ecosystems. Scientists seek improved precision of the extent of BC ecosystems; techniques to determine BC provenance; understanding of the factors that influence sequestration in BC ecosystems, with the corresponding value of BC; and the management actions that are effective in enhancing this value. Overall this overview provides a comprehensive road map for the coming decades on future research in BC science.

## Abstract (web summary)

The role of Blue Carbon in climate change mitigation and adaptation has now reached international prominence. Here the authors identified the top-ten unresolved questions in the field and find that most questions relate to the precise role blue carbon can play in mitigating climate change and the most effective management actions in maximising this.

## Introduction

Blue Carbon (BC) refers to organic carbon that is captured and stored by the oceans and coastal ecosystems, particularly by vegetated coastal ecosystems: seagrass meadows, tidal marshes, and mangrove forests. Global interest in BC is rooted in its potential to mitigate climate change while achieving co-benefits, such as coastal protection and fisheries enhancement^1–3^. BC has attracted the attention of a diverse group of actors beyond the scientific community, including conservation and private sector organizations, governments, and intergovernmental bodies committed to marine conservation and climate change mitigation and adaptation. The momentum provided by these conservation and policy actors has energized the scientific community by challenging them to address knowledge gaps and uncertainties required to inform policy and management actions.

The BC concept was introduced as a metaphor aimed at highlighting that coastal ecosystems, in addition to terrestrial forests (coined as green carbon), contribute significantly to organic carbon (C) sequestration^1^. This initial metaphor evolved to encompass strategies to mitigate and adapt to climate change through the conservation and restoration of vegetated coastal ecosystems^1,2^. As BC science consolidates as a paradigm, some aspects are still controversial; for instance, contrasting perspectives on the role of carbonate production as a component of BC^4^ and whether seaweed contributes to BC^5,6^. We propose an open discussion to refocus the current research agenda, reconcile new ideas with criticisms, and integrate those findings into a stronger scientific framework (Box 1). This effort will address the urgent need for refined understanding of the role of vegetated coastal ecosystems in climate change mitigation and adaptation.

There is, therefore, a need to establish a comprehensive research program on BC science that addresses current gaps while continuing to respond to immediate policy and managerial needs. Furthermore, this research program can inform policy directions based on new knowledge, thus playing a role in setting the management agenda and not simply responding to it. Here we identify, based on a broad effort by the leading research academics in BC science, key questions and challenges that need to be addressed to consolidate progress in BC science and inform current debate. We do so through three main steps. First, we briefly summarize the elements of BC science that represent the pillar of this research program. Second, we identify key scientific questions by first surveying the scientific community. Then we clustered these questions into common themes, which develop research goals and agendas. Last, we provide guidance as to how these questions can be best articulated into a new research agenda as a path for progress.

Box 1. Evidence underpinning the science The role of seagrasses and marine macroalgae as major C sinks in the ocean was first proposed by Smith who suggested that seagrasses and marine macroalgae were overlooked C sinks^7^; however, at the time, there was minimal uptake of the concept within climate change mitigation efforts. In 2003 the first global budget of C storage in soils of salt marshes and mangroves brought light to the importance of these coastal ocean sink. By 2005, it was shown that seagrass, mangrove, and tidal marsh sediments represent 50% of all C sequestered in marine sediments^8^. This mounting evidence for such a major role in C sequestration provided the impetus for the Blue Carbon report^1^, where the term “Blue Carbon” was first coined, and that led to the development of international and national BC initiatives (e.g., http://thebluecarboninitiative.org). This led to research efforts to propose emissions factors from loss and restoration of BC ecosystems for C accounting^9^, provide empirical evidence of emissions following disturbance and C drawdown from restoration^10,11,12^, map the C density of mangrove soils globally^13^, and explore the potential of BC ecosystems to support climate-change adaptation^2^.

## Scientists’ perspectives on the 10 key fundamental questions in BC science

We identified and selected scientists from among the leading and senior authors of the 50 most-cited papers on BC science (ISI Web of Science access date 22 June 2017), together with the participants in a workshop on BC organized at King Abdullah University of Science and Technology, Saudi Arabia, in March 2017. We did not attempt to identify any scientists’ area of specialisation to avoid bias. Among these authors, we surveyed those affiliated with academic or research institutions. A group of 50 scientists were asked to contribute from their perspective the top pending questions (up to 10) in BC science. Specifically, the invitees were asked to “Email your ten most important questions (or fewer) relevant to improving our understanding of blue carbon science and its application to climate change mitigation”. We did not ask scientists to prioritise their questions, or target any particular geographical area, but we did ask them to focus on mangrove, tidal marsh, macroalgal, and seagrass ecosystems. The answers received (35 total respondents, see Supplementary Note 1) and were then clustered into ten themes (by grouping questions that were similar) that were subsequently articulated into individual, overarching research questions:

**Q1. How does climate change impact carbon accumulation in mature Blue Carbon ecosystems and during their restoration?**

The impacts of climate change on BC ecosystems and their C stocks are dependent on the exposure to climate change factors. This is influenced by both the frequency and intensity of stressors, and the sensitivity and resilience of the ecosystem^14^. Question 1 reflects uncertainties associated with the rate and magnitude of climate change^15–17^ as well as uncertainties about the impacts of climate change on current and restored BC ecosystems, their rates of C sequestration and the stability of C stocks, which are likely to vary with past sea level history^18^, over geographic locations, among BC ecosystems, and within ecosystems.

BC ecosystems mainly occupy the intertidal and shallow water environments, where their distribution, productivity and rates of vertical accretion of soils are strongly influenced by sea level^19,20^ and the space available to accumulate sediment^21^. Thus, sea level rise ranks among the most important factors that will influence future BC stocks and sequestration. Sea level rise can result in BC gains, with increasing landward areal extent of ecosystems where possible^22^, and enhanced vertical accretion of sediments and C stocks^18,23^; and losses, with losses of ecosystem extent^24^, failure of restoration^25^, remineralization of stored organic matter^26^ that result in greenhouse gas emissions to the atmosphere (Table 1). Intense storms^17^, marine heat waves^, 27^, elevated CO_2_^28^, and altered availability of freshwater^29^ have also all been implicated as important factors affecting the distribution, productivity, community composition and C sequestration of BC ecosystems over a range of locations (Table 1). Geographic variation in exposure to climate change is high. Rates of sea level rise and land subsidence^30^, which enhances relative rates of sea level rise, vary geographically^18^. Additionally, rates of temperature change and changes in the frequency of intense storms and rainfall vary regionally^15–17^. Geomorphic models have provided first pass assessments of the global vulnerability of BC ecosystems to sea level rise^20,31^, and for restoration success^32^, but local scale descriptors of changes in exposure of BC ecosystems to climate change and impacts on C stocks are often incomplete or missing. For instance, storm associated waves are important for determining the persistence and recruitment of BC ecosystem^33^, yet local assessments are not widely available.

**Table 1:** Examples of gains and losses for BC stocks with a range of climate change factors

Ecosystem	Sea level rise	Extreme storms	Higher temperatures	Extra CO_2_	Altered precipitation
Mangrove	**Landward expansion increases area and C stocks** *Losses of low intertidal forests and coastal squeeze could reduce C stocks* **Increasing accommodation space increases C sequestration**	*Canopy damage, reduced recruitment and soil subsidence resulting in losses of C stocks* **Soil elevation gains due to sediment deposition increasing C stocks and, reducing effects of sea level rise**	Minimal impacts anticipated, although increased decomposition of soil C possible Poleward spread of mangrove forests at expense of tidal marshes increases C stocks Change in dominant species could influence C sequestration	An increase in atmospheric CO_2_ benefits plant productivity of some species which could alter C stocks	*Canopy dieback due to drought* *Losses of C stocks due to remineralization and reduced productivity* **Increased rainfall may result in increased productivity and C sequestration**
Tidal Marsh	**Landward expansion increased area and C stocks** *Losses of low intertidal marsh and coastal squeeze could reduce C stocks* **Increasing accommodation space increases C sequestration**	Loss of marsh area and C stocks **Enhanced sedimentation and soil elevation increasing C stocks and, reducing effects of sea level rise**	Increased temperatures may increase decomposition of soil organic matter, but offset by increased productivity of tidal marsh vegetation Poleward expansion of mangroves will replace tidal marsh and increase C storage Poleward expansion of bioturbators, may decrease soil C stocks	An increase in atmospheric CO_2_ benefits plant productivity of some species which could alter C stocks	*Reduced above and belowground production due to drought reducing C sequestration* Possible losses of C stocks due to remineralization Impact could be greater in areas that already have scarce or variable rainfall
Seagrass	*Loss of deep water seagrass* **Landward migration in areas where seawater floods the land (into mangrove or tidal marsh ecosystem)**	*Some extreme storms cause the erosion of seagrasses and loss of seagrass C stocks but some seagrass species are resistant to these major events* Flood events associated with extreme rainfall may result in mortality, but could also increase sediment accretion and C sequestration	*Thermal die-offs leading to losses of C stocks* Species turnover **Colonization of new poleward regions** **Increased productivity**	**An increase in dissolved inorganic C benefits plant productivity increasing C stocks** *Ocean acidification leads to loss of seagrass biodiversity, decreasing C stocks*	*Most seagrasses are tolerant of acute low salinity events associated with high rainfall, but some are negatively affected and potential interactions with disease may lead to losses of C stocks* **Reduced rainfall increases light availability which increases productivity and C sequestration**
Seaweed	*Loss of deep water seaweeds * **Seaweeds are expected to colonise hard substrata that become flooded, increasing C stocks**	*Reduces seaweed cover, but could lead to sequestration of C stocks as detritus sinks*	*Major retraction in kelp forest C stores at non-polar range edges;* **Expected expansion at polar range edges.**	Increased biomass and productivity of kelp where water temperatures remain cool enough	Little effect overall Regional effects on seaweed flora in areas with high land run off/rivers

Bold text indicate potential positive effects on BC stocks, italic text indicate negative effects with roman text indicating where effects could be positive or negative

Responses of adjacent ecosystems to climate change may influence the exposure and sensitivity of BC ecosystems and their C stocks to climate change. For example, degradation of coral reefs could increase wave heights within lagoons which may lead to losses of seagrass or mangroves within lagoons with rising sea levels as waves increase^34^, or decreases of carbonate sediments due to ocean acidification, may reduce the ability of some BC ecosystems to keep up with sea level rise^35^. Additionally, the sensitivity of BC ecosystems to climate change is also likely influenced by human activities in the coastal zone. For example, deterioration in water quality may increase the impacts of sea level rise on seagrass^36^ and decreased sedimentation from damming of rivers, hydrological modifications and presence of seawalls may negatively affect BC stocks in mangroves and tidal marshes^20,31^.

**Q2. How does disturbance affect the burial fate of Blue Carbon?**

The effect of disturbance on BC production and storage has become a topic of intense interest because of an increasing desire to protect or enhance this climate-related ecosystem service. There are three key issues, all beginning to be addressed by BC researchers, but requiring further study: (1) the depth in the soil profile to which the disturbance propagates, (2) the proportion of disturbed C that is lost as CO_2_, and (3) the extent to which issues 1 and 2 are context dependent. The first global estimates of potential losses of BC resulting from anthropogenic disturbance combined changes in the global distribution of BC ecosystems with simple estimates of conversion (remineralisation) of stored BC per unit area^37^. The estimated annual CO_2_ emission from the disturbance of BC ecosystems was estimated at 0.45 Petagrams CO_2_ globally^37^. The generalised assumptions necessary for such global assessments—e.g., remineralization within only the top 1 m of soil, and 100% loss of BC—provide little guidance at a local management scale and gloss over the variability of effects from different disturbance types^38^. This deficiency has led to a more nuanced theoretical framework accounting for the intensity of disturbance, especially whether the disturbance affects only the habitat-forming plant (e.g., clearing, eutrophication, light reduction, toxicity) or whether it also disturbs the soil (e.g., erosion, digging, reclamation)^39,40^. The duration of disturbance is another important predictor of disturbance effects on BC remineralisation because, over time, more soil BC is exposed to an oxic environment^41^.

We have a nascent understanding of the processes by which natural and human disturbances alter C decomposition. Die-off of below-ground roots and rhizomes in tidal marshes, for example, changes the chemical composition of BC and associated microbial assemblages, subsequently increasing decomposition and decreasing stored C (by up to 90% (ref. ^42^)). In seagrass ecosystems, exposing deeply buried sediments to oxygen triggered microbial breakdown of ancient BC^43^. At this stage, there is some evidence that disturbances can diminish BC stocks, for example: oil spills^44^, seasonal wrack deposition^42^, aquaculture^45^, eutrophication^46^, altered tidal flows^46^, and harvesting of fisheries resources^38,47^. Such knowledge is key for the construction of Emissions Factors for modelling. But examples in the literature are often specific for a particular disturbance or ecosystem setting, and do not yet offer the generalised understanding necessary to build a comprehensive framework guiding management projects. Finally, although there is widespread agreement that a changing climate directly affects BC production and storage, we recommend a clearer focus on the interacting effects of climate and direct anthropogenic disturbances.

**Q3. What is the global importance of macroalgae, including calcifying algae, as Blue Carbon sinks/donors?**

Macroalgae are highly productive (Table 2) and have the largest global area of any vegetated coastal ecosystem^48^. Yet only in a relatively few cases have macroalgae been included in BC assessments. Unlike angiosperms, which grow on depositional soils^2^, macroalgae generally grow on hard or sandy substrata that have no or only limited C burial potential^6^. However, a recent meta-analysis has estimated that macroalgae growing in soft sediments have a global C burial rate of 6.2 Tg C yr^−1^ (ref. ^6^), which is comparable to the lower range of estimates for tidal marshes. Furthermore, several studies show that macroalgae act as C donors^3,6,49–51^, where detached macroalgae are transported by currents, and deposited in C sinks beyond macroalgae habitats. Recent first-order estimates have suggested that up to 14 Tg C yr^−1^ of macroalgae-derived particulate organic C is buried in shelf sediments and an additional 153 Tg C yr^−1^ is sequestered in the deep ocean^6^. These calculations suggest that macroalgae may be supporting higher global C burial rates than seagrass, tidal marshes, and mangroves combined. This research highlights that if we are to incorporate macroalgal systems into BC assessments we need a better understanding of the fate of C originating from these systems. Furthermore, if we are to scale up from local measurements of C-sequestration to the global level, more refined estimates of the global surface area of macroalgal-dominated systems are needed.

**Table 2:** Estimates of global net primary productivity, CO_2_ release from calcification and C sequestration (Tg C yr^−1^) for three benthic marine systems

System	Global CO_2_ (as C) fixation in NPP	Global CO_2_ (as C) release from calcification, assuming 0.6 CO_2_-C per CaCO_3_-C produced	Global net organic C assimilation = NPP minus C as CO_2_ produced in calcification	Global C sequestration	References
Benthic macroalgae (calcified and uncalcified)	960–2000	–	–	60–1400	Charpy-Roubard & Sournia^71^; Krause-Jensen & Duarte^6^; Duarte^49^; Raven^50^
Calcified coralline red algae	720	120	600	–	Van den Heijden & Kamenos^53^, who do not mention CO_2_ release from CaCO_3_ formation
Coral reefs	0	84–840	84–840	0^a^	Ware et al.^150^; Smith & Mackenzie^151^

^a^Assuming CaCO_3_ ultimately sinks below the lysocline, where CaCO_3_ dissolves, and upwelling ultimately (10^2^–10^3^ years) brings the resulting HCO_3_^−^ back to the sea surface

Most estimates of C-sequestration by marine vegetated ecosystems refer solely to organic C even though calcifying organisms are also important components of such ecosystems^52^. For calcifying algae, whether they serve as C-sinks or sources is debated^4^, especially where calcifying organisms form and become buried within seagrass meadows^4,5^. Carbonate production results in the release of 0.6 mol of CO_2_ per mol of CaCO_3_ precipitated^53^, suggesting that calcifying algae are sources of CO_2_ that counteract C-sequestration in these ecosystems. However, co-deposition of organic and inorganic C may also have interacting effects on C-sequestration^4^. Carbonate may help protect and consolidate organic C sediment deposits, and CO_2_ release from mineralization of organic matter may stimulate carbonate dissolution and hence, CO_2_ removal^48,53,54^. Burial of inorganic carbon in seagrass and mangrove ecosystems is also to a large extent supported by inputs from adjacent ecosystems rather than by local calcification. Furthermore, mass balances highlight that such Blue Carbon ecosystems are sites of net CaCO_3_ dissolution^54^. More studies are needed to assess the net effect of organic and inorganic C deposition on C sequestration in calcifying systems.

**Q4. What is the global extent and temporal distribution of BC ecosystems?**

Our attempts to upscale BC estimates and model changes across large spatial and temporal scales is hindered by poor knowledge of their current and recent-past global distributions. The best constrained areal estimates exist for mangroves, which occur in tropical and subtropical regions, generally where winter seawater isotherms exceed 20 °C^55^. Overall, the global spatial extent of mangroves, and patterns and drivers of their temporal change, are relatively well understood, especially when compared with other BC ecosystems. Still, Giri et al.^56^ estimated a global area of mangroves of ca. 140,000 km^2^ in the year 2000 and Hamilton and Casey^57^ 83,495 km^2^ in 2000 and 81,849 km^2^ in 2012. Both studies used Landsat data but different methodologies. Mangroves occur in 118 countries worldwide, but ~75% of total coverage is located within just 15 countries, with ~23% found in Indonesia alone^56^. Total mangrove extent during the second half of the 20th century declined at rates 1–3% yr^−1^ mainly due to aquaculture, land use change and land reclamation^58^. There are uncertainties in the area of mangrove that are scrub forms and which are therefore often not considered as forests despite their importance in arid and oligotrophic settings and often their large soil C stocks^59,60^. Since the beginning of the 21st century, mangrove loss rates are 0.16–0.39% yr^−1^ (ref. ^57^), probably reflecting changes in aquaculture and conservation efforts.

Tidal marshes are primarily found in estuaries along coasts of Arctic, temperate and subtropical coastal lagoons, embayments, and low-energy open coasts, although they also occur in some tropical regions^61^. Woodwell et al.^62^ estimated global tidal marsh extent of 380,000 km^2^ using the fraction of global coastline occupied by estuaries and the assumption that ~20% of estuaries supported tidal marshes^48^. However, tidal marsh area has been mapped in only 43 countries (yielding a total habitat extent of ca. 55,000 km^2^), which represents just 14% of the potential global area^63^. Tidal marsh extent is well documented for Canada, Europe, USA, South Africa and Australia^63–65^ but remains unknown to a large extent in regions, including Northern Russia and South America. An historical assessment of 12 estuaries and coastal seas worldwide indicated that >60% of wetland coverage has been lost^66^ mostly due to changes in land use, coastal transformation and land reclamation^61^. The minimum global rate of loss of tidal marsh area is estimated at 1–2% yr^−1^ (ref. ^67^).

Despite the widespread occurrence of seagrass across both temperate and tropical regions, the global extent of seagrass area is poorly estimated^48^. The total global area was recently updated to 350,000 km^2^ (ref. ^68^), although estimates range from 300,000 (ref.) to 600,000 km^2^ (ref. ^69^), with a potential habitable area for seagrass of 4.32 million km^2^ (ref. ^70^). Available distribution data are geographically and historically biased, reflecting the imbalance in research effort among regions^71^, and most data have been collected since the 1980s^72^. The total global seagrass area has decreased by ~29% since first reported in 1879—with ~7-fold faster rates of decline since 1990 (ref. ^72^)—due to a combination of natural causes, coastal anthropogenic pressure and climate change^73^.

Producing accurate estimates of the global extent of BC ecosystems is therefore a prerequisite to assess their contribution in the global carbon cycle. In addition, given the fast rate of decline reported for many BC ecosystems, regular revision of these estimates is needed to track any changes in their global extent and importance. Extensive mapping, with particular focus on understudied areas that may support critical BC ecosystems, that combines acoustic (i.e., side scan sonar and multi-beam eco-sounder) and optical (i.e., aerial photography and satellite images) remote sensing techniques with ground truthing (by scuba diving or video images) should be undertaken to map and monitor their extent and relative change over time^74^.

**Q5. How do organic and inorganic carbon cycles affect net CO**_**2**_**flux?**

Even though BC ecosystems are significant C_org_ reservoirs, depending on C_org_ and C_inorg_ dynamics they could also be net emitters of CO_2_ to the atmosphere through air-water CO_2_ gas exchange^75^. For instance, in submerged BC ecosystems (i.e., seagrasses), C_org_ storage is not directly linked with the removal of atmospheric CO_2_ because the water column separates the atmosphere from benthic systems. BC science gaps exist in complex inorganic and organic biogeochemical processes occurring within the water column and determining CO_2_ sequestration functioning.

**Fig. 1:**
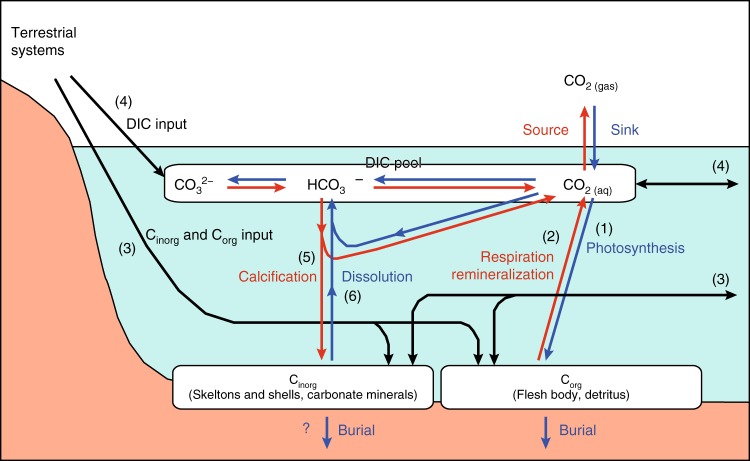
Conceptual diagram showing the biogeochemistry of carbon associated with air-water CO_2_ exchanges. Blue lines indicate the processes that enhance the uptake of atmospheric CO_2_, and red lines indicate those that enhance the emission of CO_2_ into the atmosphere. The CO_2_ concentration in surface water is primarily responsible for determining the direction of the flux. The concentration of surface water CO_2_ is determined by carbonate equilibrium in dissolved inorganic carbon (DIC) and affected by net ecosystem production (the balance of photosynthesis, respiration, and remineralization), which directly regulate DIC (1 and 2), allochthonous particulate and dissolved organic carbon (C_org_), particulate inorganic carbon (C_inorg_), and DIC inputs from terrestrial systems and coastal oceans (3 and 4), net ecosystem C_inorg_ production (the balance of calcification and dissolution), directly regulating both DIC and total alkalinity (TA) (5, 6), and temperature (solubility of CO_2_). Calcification produces CO_2_ with a ratio (released CO_2_/precipitated C_inorg_) of approximately 0.6 in normal seawater^54^

Photosynthesis lowers the CO_2_ concentration in surface water as dissolved inorganic C (DIC) is incorporated into C_org_ ((1) in Fig. 1), and respiration and remineralization increases the CO_2_ concentration ((2) in Fig. 1). Net autotrophic ecosystems would lower surface water CO_2_ concentration and be a direct sink for atmospheric CO_2_^76,77^. Lowering of surface water CO_2_ concentration is facilitated if allochthonous C_org_ ((3) in Fig. 1) and DIC inputs ((4) in Fig. 1) are low. Reactions of the inorganic C (C_inorg_) cycle can also change the CO_2_ concentration in surface water and therefore influence net exchange of CO_2_ with the atmosphere^4,5,78^. Formation of calcium carbonate minerals (calcification) results in an increase of CO_2_ in the water column ((5) in Fig. 1) while dissolution of carbonate minerals decreases CO_2_ ((6) in Fig. 1). These processes may critically affect air–water CO_2_ gas exchange. Although recent studies related to the role of BC in climate change mitigation are beginning to address the abundance and burial rate of C_inorg_ in soils^4,5,54,78–80^, studies investigating the full suite of key processes for air–water CO_2_ fluxes, such as carbonate chemistry and C_org_ dynamics in shallow coastal waters and sediments, are still scarce (but see^76,77,81,82^). In particular, relevance of carbonate chemistry to the overall spatio-temporal dynamics of C_org_ and C_inorg_ pools and fluxes (e.g., origin, fate, abundance, rate, interactions) and air–water CO_2_ fluxes is largely uncertain for BC ecosystems^4^.

Therefore, in addition to C_org_ related processes occurring in sediments and vegetation, future BC science should also quantify other key processes, such as air-water CO_2_ fluxes and C_org_ and C_inorg_ dynamics in water, to fully understand the role of BC ecosystems in climate change mitigation^83^.

**Q6. How can organic matter sources be estimated in BC sediments?**

Coastal ecosystems, mangroves, seagrasses and tidal marshes, occupy the land-sea interface and are subject to convergent inputs of organic matter from terrestrial and oceanic sources as well as transfers to and from nearby ecosystems^84^. However, the most basic requirement of quantifying organic matter inputs, and differentiating between allochthonous and autochthonous sources of C_org_, remains a challenge. This limitation has particular relevance because of interest in financing the restoration of coastal ecosystems through the sale of BC offset-credits^85^. Policy frameworks such as the Verified Carbon Standard Methodology VM0033 (ref. ^86^) stipulate that offset-credits are not allocated under the framework for allochthonous C_org_ because of the risk of duplicating C sequestration gains that may have been accounted for in adjacent ecosystems. New methods are emerging that have greater potential to quantify the contribution of different primary producers to sedimentary organic carbon in marine ecosystems^87^.

Natural abundance of stable isotopes, most commonly ^13^C, ^15^N and ^34^S, have been used to trace and quantify allochthonous and autochthonous C_org_ sources and their relative contributions to carbon burial. The costs are low, the methodology for sample preparation and analysis is relatively easy and the validity of the technique has been widely, and generally successfully tested^88^. However, the diversity of organic matter inputs can result in complex mixtures of C_org_ that are not well resolved based on the isotopic separation of the sources. Isotopic values of different species may be similar, or may vary within the same species with microhabitats, seasons, growth cycle or tissue type^89,90^.

The use of bulk stable isotopes must be improved by additionally analysing individual compounds with a specific taxonomic origin. Biomarkers such as lignin, lipids, alkanes and amino acids, have proven useful for separating multiple-source inputs in coastal sediments^88,91^. Leading-edge studies, using compound-specific stable isotopes, employ both natural and radiocarbon analyses, providing the added dimension of age to taxonomic specificity^92,93^. Oxygen and hydrogen stable isotopes could also be used to improve resolving power, but up to now they have been used mainly in foodweb studies and their utility in determining sedimentary sources in coastal systems still needs to be validated^87^. Studies using both bulk and compound-specific isotopes must consider how decomposition may alter species-specific signatures^89,90,94^ Other, alternative fingerprinting techniques are emerging. The deliberate stable isotope labelling of organic matter and tracing its fate is a powerful approach that overcomes some of the limitations of natural abundance studies (e.g., source overlap), but has only looked at short-term C_org_ burial to-date^95^. The use of environmental DNA (eDNA) has been used to describe community composition in marine systems, but the potential to quantify the taxonomic proportions of plant sources in sediments has rarely been tested^87,96^.

Overall, projects using ^13^C and ^15^N stable isotopes will likely continue to dominate the investigation of organic matter sources, especially in simple two end member systems. While there is a growing suite of organic matter tracers, the ability to distinguish between specific blue carbon sources such as marsh vegetation and seagrass still remains a challenge. Sample size requirement, analytical time and cost implications, will be crucial in the selection of the most appropriate tracers for the characterisation and quantification of the molecular complexity in blue carbon sediments. In general, applications of most compound specific tracers have focused on environments other than those supporting blue carbon ecosystems^88,93,97^, and more work is needed to apply the same research tools to these systems. We recommend, wherever possible, that complementary methods such as compound-specific isotopes and eDNA that take advantage of methodological advances in distinguishing species contributions, be used in conjunction with bulk isotopes.

**Q7. What factors influence BC burial rates?**

BC ecosystems have an order of magnitude greater C burial rates than terrestrial ecosystems^3^. This high BC burial rate is a product of multiple processes that affect: the mass of C produced and its availability for burial; its sedimentation; and its subsequent preservation. A host of interacting biological, biogeochemical and physical factors, as well as natural and anthropogenic disturbance (see Q2), affect these processes. With respect to biological factors, it remains unclear how primary producer diversity and traits (e.g., biochemical composition, productivity, size and biomass allocation) influence BC^98,99^. However, it is likely that the suite of macrophytes present in BC ecosystems is critical to the mass of C available to be captured and preserved (as suggested for tidal marshes^100^). Equally, it is uncertain how fauna influence the production, accumulation or preservation of C_org_ via top-down processes such as herbivory^38,101–103^. Similarly, predators can regulate biomass, persistence and recovery of seagrasses, marshes and mangroves by triggering trophic cascades^38^. In addition, the functional diversity and activity of the microbial decomposer community, and how they vary with depth and over time, is only just beginning to be examined^104^ and will need to be linked to BC burial rates. Most likely this microbial community will be more important in defining the fate of C_org_ entering BC soils than its production and sedimentation.

The general effects of hydrodynamics on carbon sequestration in BC ecosystems are understood, yet there is much we still do not understand which could explain the variability in sequestration we see across BC ecosystems. We know that hydrodynamics, mediated by biological properties of BC ecosystems (e.g., canopy size and structure), affect particle trapping^105–107^ and, presumably, C_org_ sedimentation rates. For example, increasing density of mangrove stands positively affects affect wave attenuation, enhancing the accumulation of fine grained material^108^, which promotes C_org_ accumulation (silts and clays retain more C_org_ than sands^109,110^. However, significant variation in soil C_org_ has been observed within seagrass meadow^111^, pointing to complex canopy-hydrodynamic interactions which we do not understand but which could affect our ability to develop robust estimates of meadow-scale BC burial. For example, a study of restored seagrass meadow found strong positive correlations between C_org_ stocks and edge proximity leading to gradients in carbon stocks at scales of >1 km^112^. Elsewhere, flexible canopies have been shown to interact with wave dynamics, increasing turbulence near the sediment surface^113^. This could explain the loss of fine sediments, and presumably C_org_, in low shoot density meadows compared to high density meadows^114^, with implications for carbon sequestration over time following restoration of BC ecosystems and the development of canopy density. Because these types of hydrodynamic interaction can affect the spatial and temporal patterns in carbon accumulation they need to be better understood in order to design stock and accumulation assessments and to predict the temporal development of stocks following management actions.

The basic biogeochemical controls on C_org_ accumulation within soils are understood (e.g., biochemical nature of the C_org_ inputs which vary among primary producers^115–117^ and the chemistry of their decomposition products)^110^, but it remains unclear what controls the stability of stored C_org_ in BC soils and whether these factors vary across ecosystems or under different environmental conditions (incl. disturbance). With the exception of one recent paper^43^, we know little about the C_org_ -mineral associations in BC ecosystems, how these affect the recalcitrance of soil C_org_ or whether specific forms are protected more by this mechanism than others, though this is clearly the case in other ecosystems^118–120^. Undoubtedly the anaerobic character of BC soils places a significant control on in situ rates of C_org_ decomposition and remineralisation. However, the time organic materials are exposed to oxygen before entering the anaerobic zone of BC soils will impact the quantity and nature of C_org_ as will the redox potential reached within the soil. The amount of time organic matter is exposed to oxygen explains the observation that C_org_ concentrations in tidal marshes globally are higher on coastlines where relative sea level rise has been rapid compared to those where sea level has been relatively stable^18^. Moreover, exposure of BC to oxygen has been recently shown trigger microbial attack, even ancient (5000-year-old) and chemically recalcitrant BC^43^. Enhancing our understanding of oxygen exposure times and critical redox potentials will help explain variations in C_org_ accumulation rates and preservation within different BC ecosystems.

From the above, there is increasing evidence that we do not understand the complex interactions among influencing environmental factors well enough to predict likely C_org_ stocks in soils, including temperature, hydrodynamic, geomorphic and hydrologic factors that can affect biogeochemical processes or mediate biological processes, and this leads to apparent contradictions. For example, the influence of nutrient availability on C_org_ stocks is unclear with one study reporting an increase in soil C_org_ stocks along a gradient of increasing phosphate availability^121^, another reporting no effect^122^, and yet others^121,123^ finding that increasing nutrient availability led to lower soil C_org_. Some empirical studies have examined interactive effects or evoked them to explain difference in C_org_ stock^101,124,125^. However, these studies are rare and limited by the complexity or the interactions being examined. We conclude that gaining insights into these interactive effects is more likely to be advanced through modelling approaches.

**Q8. What is the net flux of greenhouse gases between Blue Carbon ecosystems and the atmosphere?**

BC ecosystems are generally substantial sources or sinks of greenhouse gases (GHGs) (CO_2_, CH_4_, N_2_O), though we cannot construct accurate global BC budgets due to uncertainties in net fluxes. The C budget is best constrained for mangroves, with mangroves globally taking up 700 Tg C yr^−1^ through Gross Primary Production, and respiring 525 Tg C yr^−1^ (75%) back to the atmosphere as CO_2_^126^. However, large uncertainty exists in budgets due to poorly constrained mineralization pathways linked to CO_2_ efflux^119^.

We lack robust global C budgets for other BC ecosystems due to insufficient empirical evidence^127^. For example, while we have estimated global soil C_org_ stocks^128^ and accumulation rates for seagrasses, this is insufficient to create a budget^129^ because we lack representative data on community metabolism and GHG fluxes, particularly for CH_4_ and N_2_O emissions. Thus, we need to better quantify sink/source balances, e.g., the net balance between primary production vs. emissions from ecosystem degradation and pelagic, benthic, forest floor and canopy respiration^126^. We also need to understand how source/sink dynamics change budgets over time and how environmental parameters affect GHG fluxes^129,130^, allowing us to estimate thresholds that flip BC ecosystems from GHG sinks to sources.

Budgets generally focus on CO_2_ fluxes, though we must better understand fluxes of other GHGs such as CH_4_ and N_2_O, and their contribution to the global BC budget^131^. Global estimates show that CH_4_ emissions can offset C burial in mangroves by 20% because CH_4_ has a higher global warming potential than CO_2_ on a per molecule basis^132^. CH_4_ emissions may also offset C burial in seagrasses, though these estimates have not been made. In contrast, some mangroves are N_2_O sinks^133^ which would enhance the value of the C burial as a means to mitigate climate change. Overall, CH_4_ and N_2_O biogeochemistry is understudied in BC ecosystems.

Finally, we must understand how GHG fluxes change as BC ecosystems replace each other, such as when mangroves expand onto marshes at their latitudinal limits^134^, or are planted on seagrass meadows in Southeast Asia. We also need to understand how emissions may change with loss of BC ecosystems. For example, it has been coarsely estimated that a 50% loss of seagrass would result in a global reduction in N_2_O emissions of 0.012 Tg N_2_O-N yr^−1^ and a 50% loss of mangroves would result in a global reduction in emissions of 0.017 Tg N_2_O-N yr^−1^ (ref. ^130^).

**Q9. How can we reduce uncertainties in the valuations of Blue Carbon?**

**Fig. 2:**
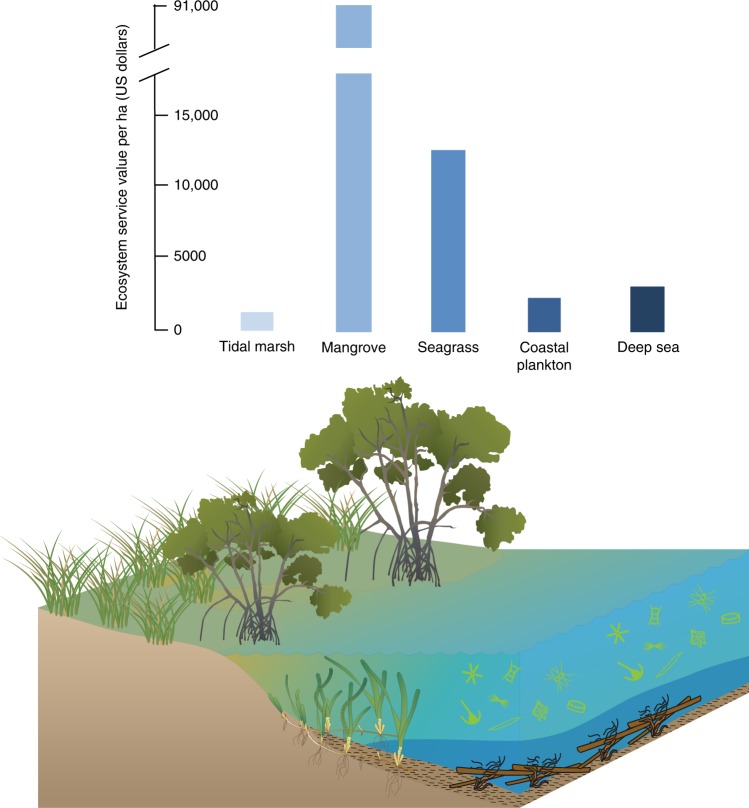
Estimates of the economic value of blue carbon ecosystems per hectare. Data from ref. ^1^ and references therein. Symbols and images are courtesy of the Integration and Application Network, University of Maryland Center for Environmental Science (ian.umces.edu/symbols/)

Studies into BC increasingly include a valuation aspect, focussed on coastal sites^135^ but more recently also including offshore sites^136^, showing a range of values for different ecosystems as depicted in Fig. 2. Differences in values are driven by differences in BC sequestration and storage capacity and/or potential avoided emissions through conservation and restoration of ecosystems. There is also variation in BC values due to uncertainties in the calculation of C sequestration and permanence of C storage, as is required for valuation. The wide range of C valuation methods, including social costs of C^111^, marginal abatement costs^112^, and C market prices, also enhances the uncertainty and variation in valuation estimates.

Valuation of BC enables its inclusion in policy and management narratives^113^, facilitating the comparison of future socio-economic scenarios, including mitigation and adaptation interventions^137^, and raises conservation interests as an approach to mitigate climate change and offset CO_2_ emissions^2^. For example, BC budgets can be incorporated into national greenhouse gas inventories^138^. Alternatively, demonstrable gains in C sequestration and/or avoided emissions through conservation and restoration activities can be credited within voluntary C markets or through the Clean Development Mechanism of the United Nations Framework Convention on Climate Change (UNFCCC)^86^. Voluntary market methodologies for BC ecosystems have been released within the American Carbon Registry^139^ and within the Verified Carbon Standard^86^, while some countries are developing BC-focussed climate change mitigation schemes that provide economic incentives. However, on the international scale, BC ecosystems have previously not been consistently incorporated into frameworks for climate change mitigation that offer economic reward for the conservation of C sinks, such as the REDD + program^140^, possibly as there was insufficient information for its inclusion. Avoiding degradation of mangroves, tidal marshes and seagrasses could globally offer up to 1.02 Pg CO_2_-e yr^−1^ in avoided emissions^37^. Developing countries with BC resources have the opportunity to use BC for the NDC, for example Indonesia, where BC contribution to reduce emissions could be as much as 0.2 Pg CO_2_-e yr^−1^ or 30% of national land-based emission while mangrove deforestation only contributes to 6% of national deforestation^141^.

To reduce uncertainty in BC values and encourage use of values in future policy and management, we recommend improved interdisciplinary research, combining ecological and economic disciplines to develop standardised approaches to improve confidence in the valuation of BC. Ideally this should be undertaken alongside studies which recognise the additional values of conserving BC ecosystems, for example the benefits generated from fisheries enhancement, nutrient cycling, support to coastal communities and their livelihoods^2^ and coastal protection, which is considered a cost-effective method compared to hard engineering solutions^142^.

**Q10. What management actions best maintain and promote Blue Carbon sequestration?**

Research over the past decade has improved estimates of C dynamics at a range of spatial scales. This has enabled modelling of potential emissions from the conversion of seagrass, mangrove and tidal marsh to other uses^41^, and estimates of rates of and hotspots for CO_2_ emissions resulting from ecosystem loss. The development of policy, implementation of management actions and the demonstration of BC benefits (including payments), however, are still in their infancy.

There are three broad management approaches to enhance C mitigation by BC ecosystems: preservation, restoration and creation. Preserving ecosystem extent and quality—for example, through legislative protection and/or supporting alternative livelihoods—has the two-fold benefit of avoiding the remineralisation of historically sequestered C, while also protecting future sequestration capacity. Preservation may include direct or indirect approaches to maintain or enhance biogeochemical processes, such as sedimentation and water supply^46^. Restoration pertains to a range of activities seeking to improve biophysical and geochemical processes—and therefore sequestration capacity—in BC ecosystems. Examples include passive and/or active reforestation of logged and degraded mangrove forests^143^; earthwork interventions to return aquaculture ponds to mangrove ecosystems^141^; and the restoration of hydrology to drained coastal floodplains^144^. Managed realignment is a particular option for creating or restoring tidal marshes as part of a strategy to achieve sustainable coastal flood defence together with the provision of other services, including C benefits^145^; other similar options include: regulated tidal exchange^131^ and beneficial use of dredged material^146^. Although restoration may re-establish C sequestration processes, it is important to note that it may not prevent large amounts of fossil C being lost following future disturbance or intervention. ‘No net loss’ policies have been now developed and applied to wetland ecosystems in many countries (e.g., USA and EU). These generally imply the creation of BC ecosystems to replace those lost through development. Such approaches should be treated with caution, however, since there is confusion about terminology^141^, lack of enforcement and limited capacity to recreate the qualities of pristine sites.

Tools for the accounting and crediting of C payments now exist for coastal wetland conservation, restoration and creation under the voluntary C market^86,147^. Several small-scale projects (e.g., Mikoko Pamoja in Kenya) are now using these frameworks to generate C credits with others projects in development^148^. Few jurisdictions have adopted their own mechanisms for the accounting and/or trading of BC, though some have undertaken preliminary research to identify BC policy opportunities^149^.

Technical, financial and policy barriers remain before local initiatives can be scaled-up to make large impacts—such as through national REDD + initiatives. Significant barriers include: biases in the geographic coverage of data; approaches for robust, site-specific assessment and prediction of some C pools (e.g., below-ground C and atmospheric emissions); high transaction costs; and ensuring that equity and justice are achieved. In addition, most demonstrated efforts are recent actions with little quantification of C mitigation benefits (or societal outcomes) beyond the scale of a few years.

Despite such barriers, we now have the fundamental knowledge to justify the inclusion of BC protection, restoration and creation in C mitigation mechanisms. While there remain knowledge gaps—both in science, policy and governance—these will partly be addressed through the effective demonstration, monitoring and reporting of existing and new BC projects.

## Toward a research agenda on the role of vegetated coastal ecosystems on climate change mitigation and adaptation

The questions above are not short of challenges and therefore, provide ample scope for decisive experiments to be designed and conducted, current hypotheses to be rejected or consolidated and new ideas and concepts to unfold. Emerging questions that are not yet supported by robust observations and experiments, include, for example: the estimation of allochthonous C (organic and inorganic) contributions to BC, which remains challenging due to availability of markers able to quantitatively discriminate among the different carbon sources; and the net balance of GHG emissions, which remains challenging as it requires concurrent measurements across relevant time and spatial scales of all major GHGs (CO_2_, CH_4_, NO_2_), for which not a single estimate is available to-date. The core questions that capture much of current research efforts in BC science include the role of climate change on C accumulation, efforts to improve the precision of global estimates of the extent of BC ecosystems, factors that influence sequestration in BC ecosystems, with the corresponding value of BC, and the management actions that are effective in enhancing this value. The preceding text provides a summary of current research efforts and future opportunities in addressing these key questions.

Three questions are long-standing, controversial, and need resolution in order to properly constrain the BC paradigm. The first is the effect of disturbance on GHG emissions from BC ecosystems, where the initial assumption, that the top meter of the soil C stock is likely to be emitted as GHG following disturbance^37,128^, continues to be carried across papers without being challenged or verified. The second is whether macroalgae-C can be considered BC. The term BC refers to C sequestered in the oceans^1^, and the focus on seagrass, mangroves and tidal marshes is justified by the intensity of local C sequestration these ecosystems support. If macroalgae provide intense C sequestration, whether in the ecosystem or beyond, they need to be dealt with in this context. And the third controversy is whether carbonate accumulation in BC ecosystems render them potential sinks of CO_2_ following disturbance. It is clear that there are far too many key uncertainties^4^ to resolve this at the conceptual level, since empirical evidence to provide a critical test is as yet lacking. We propose that a research program including key observational and experimental tests designed to resolve the mass balance of carbonate (e.g., balance between allochthonous and autochthonous production and dissolution)—and then the coupling between BC ecosystems and the atmosphere—is needed. In the case of all three controversies, we believe that the positive approach to address these questions, is to pause the current discussion, which are largely rooted in the lack of solid, direct empirical evidence, and recognize that further science is required before any conclusion can be reached.

In summary, the overview of questions provided above portrays BC science as a vibrant field that is still far away from reaching maturity. Apparent controversies are a consequence of this lack of maturity and need to be resolved through high quality, scalable and reproducible observations and experiments. We believe the questions above inspire a multifarious research agenda that will require continued broadening the community of practice of BC science to engage scientists from different disciplines working within a wide range of ecosystems and nations.

## Supplementary information

Supplementary Information
