# Author Correction: The future of Blue Carbon science

**DOI:** 10.1038/s41467-019-13126-0

**Published:** 2019-11-08

**Authors:** Peter I. Macreadie, Andrea Anton, John A. Raven, Nicola Beaumont, Rod M. Connolly, Daniel A. Friess, Jeffrey J. Kelleway, Hilary Kennedy, Tomohiro Kuwae, Paul S. Lavery, Catherine E. Lovelock, Dan A. Smale, Eugenia T. Apostolaki, Trisha B. Atwood, Jeff Baldock, Thomas S. Bianchi, Gail L. Chmura, Bradley D. Eyre, James W. Fourqurean, Jason M. Hall-Spencer, Mark Huxham, Iris E. Hendriks, Dorte Krause-Jensen, Dan Laffoley, Tiziana Luisetti, Núria Marbà, Pere Masque, Karen J. McGlathery, J. Patrick Megonigal, Daniel Murdiyarso, Bayden D. Russell, Rui Santos, Oscar Serrano, Brian R. Silliman, Kenta Watanabe, Carlos M. Duarte

**Affiliations:** 1 Deakin University, School of Life and Environmental Sciences, Center for Integrative Ecology, Geelong, VIC 3125 Australia; 2 King Abdullah University of Science and Technology, Red Sea Research Center and Computational Bioscience Research Center, Thuwal, Saudi Arabia; 3 Division of Plant Sciences, University of Dundee at the James Hutton Institute, Invergowrie, Dundee, DD2 5DQ UK; 4 Climate Change Cluster, University of Technology Sydney, Ultimo, NSW 2007 Australia; 5 School of Biological Science, University of Western Australia, 35 Stirling Highway, Crawley, WA 6009 Australia; 6 Plymouth Marine Laboratory, Prospect Place, Plymouth, PL1 3DH UK; 7 Australian Rivers Institute—Coast & Estuaries, School of Environment and Science, Griffith University, Gold Coast, QLD 4222 Australia; 8 Department of Geography, National University of Singapore, 1 Arts Link, Singapore, 117570 Singapore; 9 School of Earth, Atmospheric and Life Sciences, University of Wollongong, Wollongong, NSW 2522 Australia; 10 School of Ocean Sciences, Bangor University, Menai bridge, Bangor, LL59 5AB UK; 11 Coastal and Estuarine Environment Research Group, Port and Airport Research Institute, 3-1-1 Nagase, Yokosuka, 239-0826 Japan; 12 School of Science, Centre for Marine Ecosystems Research, Edith Cowan University, 270 Joondalup Drive, Joondalup, WA 6027 Australia; 13 School of Biological Sciences, The University of Queensland, St Lucia, QLD 4072 Australia; 14 Marine Biological Association of the United Kingdom, Citadel Hill, Plymouth, PL1 2PB UK; 15 Institute of Oceanography, Hellenic Centre for Marine Research, PO Box 2214, 71003 Heraklion, Crete Greece; 16 Department of Watershed Sciences and Ecology Center, Utah State University, Logan, UT 84322-5210 USA; 17 CSIRO Agriculture and Food, Private Mail Bag, Glen Osmond, SA 5064 Australia; 18 Department of Geological Sciences, University of Florida, Gainesville, FL 32611-2120 USA; 19 Department of Geography, McGill University, 805 Sherbrooke St W, Montreal, QC H3A 0B9 Canada; 20 Centre for Coastal Biogeochemistry, School of Environment, Science and Engineering, Southern Cross University, Lismore, NSW 2480 Australia; 21 Department of Biological Sciences and Center for Coastal Oceans Research, Florida International University, 11200 SW8th St, Miami, FL 33199 USA; 22 School of Biological and Marine Sciences, University of Plymouth, Plymouth, UK; 23 Shimoda Marine Research Center, University of Tsukuba, Tsukuba, Japan; 24 School of Applied Sciences, Edinburgh Napier University, Edinburgh, EH11 4BN UK; 25 Global Change Research Group, IMEDEA (CSIC-UIB), Institut Mediterrani d’Estudis Avançats, Miquel Marquès 21, Esporles, 07190 Spain; 26 Department of Bioscience, Aarhus University, Vejlsøvej 25, Silkeborg, 8600 Denmark; 27 Arctic Research Centre, Department of Bioscience, Aarhus University, Ny Munkegade 114, bldg. 1540, Århus C, 8000 Denmark; 28 World Commission on Protected Areas, IUCN, Gland, Switzerland; 29 Centre for Environment, Fisheries, and Aquaculture Science, Lowestoft, UK; 30 The Oceans Institute and Department of Physics, The University of Western Australia, 35 Stirling Highway, Crawley, WA Australia; 31 Departament de Física & Institut de Ciència i Tecnologia Ambientals, Universitat Autònoma de Barcelona, Bellaterra, 08193 Spain; 32 Department of Environmental Sciences, University of Virginia, Charlotttesville, VA 22903 USA; 33 Smithsonian Environmental Research Center, 647 Contees Wharf Road, Edgewater, MD 21037 USA; 34 Center for International Forestry Research (CIFOR), Jl. CIFOR, Situgede, Bogor, 16115 Indonesia; 35 Department of Geophysics and Meteorology, Bogor Agricultural University, Kampus Darmaga, Bogor, 16680 Indonesia; 36 Swire Institute of Marine Science, School of Biological Sciences, University of Hong Kong, Hong Kong SAR, China; 37 Center of Marine Sciences, CCMAR, University of Algarve, Faro, 8005-139 Portugal; 38 Nicholas School of the Environment, Duke University, 135 Duke Marine Lab Road, Beaufort, NC 28516 USA

**Keywords:** Microbiology, Plant sciences, Biogeochemistry

Correction to: *Nature Communications* 10.1038/s41467-019-11693-w, published online 05 September 2019.

The original version of this Article contained an error in the author affiliations.

Affiliation 24 incorrectly read ‘School of Applied Sciences, Edinburgh University, Edinburgh EH11 4BN, UK’

This has now been corrected in both the PDF and HTML versions of the Article.

