# Supplementary material for: The future of Blue Carbon science
Source: Nat Commun. 2019 Sep 5;10:3998. doi: 10.1038/s41467-019-11693-w (PMC6728345; doi:10.1038/s41467-019-11693-w)
Supplement: Supplementary file 1 — Supplementary Information [file 41467_2019_11693_MOESM1_ESM.pdf]

Supplementary Note 1

List of the questions submitted by co-authors identifying perceptions of key questions in Blue Carbon science

Andrea Anton

- What are the global areas of the main Blue Carbon habitats (seaweeds, mangroves, seagrasses, and saltmarshes)?
- What are and where are the main carbon sinks for macroalgae?
- What are the carbon storage rates for macroalgae in the deep sea?

Bayden Russell

- The fate of C sequestered by macroalgae which then die on short time scales. Many are annual to less than a decade in life span and the lost carbon can then potentially re-enter the carbon system;
- Fate of C which is lost as tissue from macroalgae. Unlike terrestrial biomes where any shed carbon (e.g. leaves, other biomass) can be incorporated into the soil and therefore "locked away", the fate of this biomass is relatively unknown for macroalgal stands.
- Realistic predictions of our ability to restore habitats in the face of ongoing and persistent pollution (local to regional discharges) and increased temperatures.
- The trade-off between ongoing aquaculture development in Asia and Africa and Blue Carbon stores – currently practices are generally in conflict
- Can aquaculture be used as a Blue Carbon? What is the fate of the carbon along the consumption chain? Does this actually count towards C reduction?

Bradley Eyre

- CH4 and N2O offsets to Blue Carbon burial
- Carbonate burial offsets to Blue Carbon burial
- Autochthonous versus allochthonous carbon source contributions to Blue Carbon burial

- Area estimates of Blue Carbon burial habitats, particularly at the species level (e.g. Zostera vs Halophila) and sub-type level (e.g. river vs ocean mangroves).
- How do you upscale areas. Lots of different ways.
- Lack of burial data for Blue Carbon burial habitats, particularly replicated burial rates and at the species level (e.g. Zostera vs Halophila) and sub-type level (river vs ocean mangroves)

Brian Silliman

- How does the increasing rate of disturbance in coastal wetlands impact Blue Carbon?
- How do habitat cascades impact spatial variation in Blue Carbon storage?
- How do filter feeding bivalves regulate carbon sequestration in vegetated coastal wetlands?
- How do different types of development (reclamation, shrimp farming etc) impact Blue Carbon in mangroves?
- How does Blue Carbon storage vary with time since restoration in restored wetlands?
- What is the density-dependent impact of grazers on Blue Carbon storage?
- How do predators indirectly control Blue Carbon, and how does that vary with predator identity and density?
- How does frequent drought impact carbon storage in coastal wetland?
- How does sea level rise impact carbon storage?
- How does loss of Blue Carbon storage vary with length of disturbance event?

Carlos Duarte

- What is the area covered by seagrass and how is it distributed globally? (As the areas for mangroves and salt-marshes are now relatively well constrained).
- What is the global distribution of organic carbon density, burial rates and stocks in BC habitats? (As estimates published thus far may have been biased towards particular regions or, in the case of seagrass, upper estimates).
- What is the net balance between emissions of greenhouse gases and organic carbon burial in Blue Carbon habitats?
- How do macro algae contribute to carbon sequestration?
- What is the fate of exported production from Blue Carbon habitats, including macroalgae, where do these stocks accumulate?

- What is the role of carbonates in Blue Carbon sediments and how does it affect greenhouse accounting and organic carbon preservation?
- What is the best approach to fingerprint the contributions of different sources to Blue Carbon organic stocks?
- How should allochthonous contributions be considered in terms of greenhouse accounting?
- Does climate change affect the stability of Blue Carbon CO2 sequestration and stocks?

Catherine Lovelock

- Are Blue Carbon ecosystems "safer" or more prone to disturbance than terrestrial carbon sinks?
- What happens to Blue Carbon stocks with sea level rise?
- What is the shape of the trajectories of C sequestration upon restoration – what should those models look like? Linear for soil; exponential for biomass? How many years to reach targets?
- What is the cost/benefit of Blue Carbon projects – are they really more expensive than terrestrial ones? Which ones are and which ones are not?

Dan Friess

- Can we better quantify dissolved carbon fluxes in mangroves? A large part of the global mangrove carbon budget is unaccounted for. This may be because we have poor knowledge of dissolved flux pathways for DIC and DOC, particularly sub-surface tidal pumping and groundwater fluxes.
- What are the carbon links between Blue Carbon ecosystems? We need a better understanding of how Blue Carbon ecosystems exchange carbon between them, both spatially and through time.
- How can we better quantify the spatial distribution of soil carbon at multiple scales? This is a potential constraint to Blue Carbon Payments for Ecosystem Services (PES), and it would better incentivize conservation if we could include soil carbon stocks. However, this needs us to develop better methods to quantify soil carbon at scales larger than plot measurements.
- How can we accurately upscale estimates of soil carbon accretion? Some studies have linked measurements of soil carbon with sediment accretion rates to understand soil carbon accumulation over time. However, sediment accretion and surface elevation processes are

hugely variable across a site due to geomorphology, microtopography, disturbance etc. Our inability to measure the spatial variation of accretion constrains our current estimates of soil carbon accumulation.

- What time scales are required for restored mangroves to attain natural carbon cycling function? Through studies of forestry plantations we have an idea of how much time is required to restore some parts of the carbon budget (e.g., above-ground biomass) to a predisturbance state. However, the same cannot be said for dissolved and gaseous fluxes, and to a lesser extent soil carbon stocks.
- Can we better constrain emissions factors during land cover conversion? We now have quite a solid picture of Blue Carbon stocks in many parts of the world. However, we rarely measure the carbon stocks of alternative land uses. This information is required if we are to more accurately quantify Blue Carbon loss during habitat conversion. Emissions factors for mangroves and alternative land uses are urgently needed.
- What are the emissions from degraded mangroves? We need a better understanding of the disturbance thresholds that flip mangroves into carbon emitters, particularly soil gaseous fluxes.
- What are the governance and implementation challenges to Blue Carbon PES? Despite years of research and discussion, Blue Carbon PES is still at an embryonic stage. We need to better understand the socio-economic constraints to Blue Carbon PES implementation, how it differs between countries, and how it differs from terrestrial PES mechanisms.

Dan Laffoley

- How do Blue Carbon habitats respond to a changing terrestrial environment? (Craig Smeaton/William Austin)
- Within the sediment stores associated with Blue Carbon habitats, how should we account for terrestrial Carbon subsidies? (William Austin/Craig Smeaton)
- What role will future sea level rise play in the potential of coastal Blue Carbon habitats to sequester and store Carbon? (William Austin)
- How significant are Blue Carbon habitats in subsidizing Carbon to subtidal sediments? (William Austin)

- To what degree and geographical extent does sea grass influence the carbonate chemistry of the surrounding waters including carbon absorption as well as locally buffering ocean acidification scenarios (Dan)
- Quantification and timescales for the carbon re-release pathways for subtidal sink habitats such as sea grass and maerl as a result of impacts and damage from human activities including trawling (Dan)
- Does having a local profusion of subtidal carbon pools and sinks confer any buffering advantages down the line from effects of progressive ocean acidification (Dan)
- How does subtidal carbon pools and sinks stability interact with a warming and deoxygenating ocean? (Dan)

Daniel Smale

- What is the spatial distribution (at local to global scales) of Blue Carbon source and sink habitats and what environmental factors drive their distributions?
- What is the standing stock (above and below ground) of organic carbon (at local to global scales) in Blue Carbon habitats and what environmental factors drive variability in standing stock?
- What is the burial rate and long-term storage capacity of organic carbon (at local to global scales) within Blue Carbon habitats and what environmental factors drive variability in storage capacity?
- What is the significance of carbon donors (e.g. macroalgae) for Blue Carbon ecosystem services?
- How interconnected are Blue Carbon habitats and what processes determine transport pathways of organic carbon and source-sink connectivity?
- What is the contribution of allochthonous organic carbon (i.e. from terrestrial sources and macroalgae) to total carbon storage in Blue Carbon habitats and how does this vary spatio-temporally?
- How will climate change (i.e. ocean warming, sea level rise, increased storminess) alter the assimilation, transport, burial and storage of organic carbon in Blue Carbon habitats?
- How will climate change-carbon cycle feedbacks/interactions influence Blue Carbon services?

- How will local/regional anthropogenic stressors (e.g. physical disturbance from fishing/shipping practises, decreased water quality, coastal development and land use) alter the assimilation, transport, burial and storage of organic carbon in Blue Carbon habitats?
- What are the implications of rapid ice loss in the Arctic and Antarctic for the expansion of Blue Carbon habitats and natural carbon sequestration?
- What management actions/approaches (e.g. MPAs, fishing restrictions, land use management) best maintain and promote natural carbon sequestration? Can or should international policy frameworks be expanded to include carbon donor habitats (e.g. kelp forests) within the context of natural carbon sequestration?

Dorte Krausse-Jensen

- What is the contribution of macroalgae to carbon sequestration? - there is a need of field data on the contribution of macroalgae to sediment carbon stocks, estimates of carbon export from macroalgae etc.
- To what extent is macroalgal carbon preserved in sediments? - differences between species, habitats.
- To what extent does DOC emitted from macroalgae forests and seagrass meadows contribute to carbon sequestration?
- What is the carbon emission from eroded seagrass sediments?
- To what extent does macroalgal cultivation contribute to carbon sequestration, - e.g. to what extent and how can the seaweed biomass that is currently discarded from production be used in carbon sequestration?

Eugenia Apostolaki

- Expand the data-set of carbon sequestration, burial and storage of Blue Carbon ecosystems (different vegetation type, species and biogeographic regions)
- Assess the carbon sequestration, burial and storage of mixed vs. monospecific seagrass meadows
- Identify the environmental variables that are responsible for the variability in the carbon sink capacity of seagrass ecosystems (e.g. hydrodynamic regime, depth, temperature, sediment granulometry)

- Study the effect of direct anthropogenic impacts [e.g. eutrophication, fish farming, coastal development, mechanical damage (trawling, dredging, anchoring)] on the carbon sink capacity and burial rate in seagrass ecosystems
- Study the effect of climate change on the carbon sink and burial in seagrass ecosystems (e.g. warming, acidification, alien species invasion)
- Assess and quantify the sources of organic matter (carbon and nutrients) stored in Blue Carbon ecosystems
- Assess and quantify the fate of organic matter released after the carbon sinks get impacted or destroyed
- Evaluate the organic matter (carbon and nutrients) transferred/ exchanged between adjacent Blue Carbon ecosystems extending at different zones (e.g. supralittoral to intertidal to subtidal and salt marsh to seagrass or mangroves to seagrass)

Gail Chmura

- What is the alternate fate (in open ocean, estuaries, tidal flats) of allochthonous carbon trapped in restored salt marshes and mangroves?
- What is the rate of loss of OC or IC when BCE's are drained?
- What role does tidal amplitude play in rates of OC accumulation during restoration?
- What role does climate play in C accumulation in mature BCE's and during their restoration?

Hilary Kennedy

- Does allochthonous and autochthonous organic carbon have equal validity in assessing C stocks and accumulation?
- How important is it that coastal ecosystems store terrestrially derived soil?
- What variables need to be included to predictively model OC accumulation and storage across different environmental settings?
- How can modelling help in scaling up from local measurements to the global scale?
- What is the most effective way to measure baselines on which to base temporal (or spatial) change?
- What is the fate of autochthonous organic carbon in natural settings and soil C after vegetation loss in degraded ecosystems?

- How can we effectively map submerged C stores?
- How does carbonate production and dissolution affect carbon fluxes and storage in the short and longer term?
- When should we be measuring gas (carbon dioxide, methane and nitrous oxide), rather than solid phase, fluxes?
- How can we improve both, identification of anthropogenic from natural change, and quantification of the impact of converted, disturbed and degraded ecosystems?

Iris Hendricks

- Carbonate Question: isn´t this really a matter of time scales, whether carbonate production is a source or sink of CO2. Wouldn´t it be fascinating to determine the kind of scale that is relevant and specifically see if we can get to a working definition of when we call it sink (cliffs of Dover? - if a calcified organism is deposited and buried) or source (calcification in the water column and/or in this very moment), like a threshold time period.
- Related: how would we denominate calcifying algae? Sources or sinks? Should we measure net fluxes per species (but see next whether algae are even relevant)
- Related: should we include calcifying epiphytes in our calculations of carbon accounting?
- The Macroalgae question: eligible for carbon accounting or not? If we focus on carbon deposits no (or yes? if material gets transported to the deep sea - too early to say) but focussing on air-sea fluxes yes.
- Are points measurements of fluxes sufficient with the huge seasonal/daily variations of NPP? Are we over/underestimating fluxes this way? Again if we only focus on burial capacity less relevant
- Why does carbon need to be deposited on "millenary" scales for carbon accounting schemes? That sounds absolutely irrelevant seeing we cannot even predict how the earth will be in 100 years (i.e. changing IPCC predictions).
- How relevant is horizontal advection in carbon transport from/to vegetated ecosystems? Can we pool it as "continental shelf area"/"coastal ecosystem" and assume the transport between patches of vegetation are not relevant or is hydrodynamic transport (i.e. between coral reefs and seagrass) actually very relevant? Should the scale of the Blue Carbon initiative and regional hydrodynamics determine the importance and how to treat differences between Blue

Carbon projects of the same scale in energetically different areas (or changing wave regimes with global change - regional differences).

- Should we actively seek to sequester carbon by artificially enhancing oceans´ capacities to take up CO_2 like by olivine addition (mineral stone weathering) - much like iron fertilization in limited areas? and should this be an alternative to Blue Carbon (loss of co-benefits of vegetation) or be discouraged in favor of Blue Carbon

Jason Hall-Spencer

- How will ocean acidification and warming affect photoautotrophs and their role in the marine carbon cycle?
- Can we expect more or less carbon sequestration by marine algae and plants as CO_2 levels rise?
- What proportion of oil, gas and coal reserves has been created by each of the following - seagrasses, salt marshes, mangroves, seaweeds and phytoplankton?
- Is growing seaweeds for food a good way of taking nutrients and carbon out of waters that are polluted with fertilizers and CO_2?

Jeff Baldock

- What controls the stability of organic carbon in the soil under Blue Carbon ecosystems (Mangroves, tidal marshes, seagrasses) and do they vary across habitats or with differences in environmental conditions?
    - Input chemistry – chemical nature of the organic inputs
    - Chemistry of products of decomposition – do changes in chemical composition during decomposition infer stability
    - Decomposer community variations – does the capability exist to decompose a wide range of forms of organic matter.
    - Mineral association – how does association with minerals impact on the decomposability of organic materials in soils, does this vary with different mineralogies or surface areas, are specific forms (chemistries) of OC protected more by this mechanism than other forms.

  - Environment - O2 exclusion (biological oxygen demand, oxygen exposure time) and temperature effects as modifiers of process rates (biological and chemical)
- Rates of organic carbon input into the systems.
- Differentiating autochthonous from allochthonous contributions – probably more important in the carbon accounting realm. As far as the atmosphere is concerned, if the carbon is stabilised the atmosphere does not see it, so it should not matter what the initial source is. This then really only becomes a question associated with our attempts to ensure proper C mass balance in C accounting exercises.
- How to effectively sample to measure current stocks and stock change (issues: depth of sampling, minimising the impact of spatial variance through sampling designs to allow better isolation and detection of the temporal change).
- Development of predictive models for OC cycling in Blue Carbon systems – although understanding all the above would be required, we could get something started and modify it as understanding improves.

Jeffrey Kelleway

- What will be the fate of BC habitats and existing BC stocks under SLR?
- What will be the fate of BC habitats and existing BC stocks under warming and enhanced atmospheric CO2?
- What are the most promising restoration/creation options for C abatement and how do these vary among jurisdictions
- Can we accurately predict BC stocks, accumulation rates and/or emissions with remotely sensed data?
- What are the remineralisation rates of mangrove, tidal marsh and seagrass soils?
- What are the drivers of Blue Carbon remineralisation?

Jim Fourqurean

- what is the fate of stored Corg upon disturbance of Blue Carbon habitats?
- how important are the macrophytes in Blue Carbon habitats in the accumulation and retention of Corg in the sediments?

- how does the inorganic C cycle interact with net ecosystem metabolism to influence flux of C from Blue Carbon habitats to the atmosphere?
- will active creation or restoration of Blue Carbon habitats lead to a net increase in C uptake and storage in blue carbon ecosystems
- under what circumstances are the plant communities necessary for the retention of C in Blue Carbon ecosystems (a slight variation on my question #2)
- does disturbance of Blue Carbon habitats influence the lability of Corg stored in them?
- how much of a threat is sea level rise and climate change to the storage of C in Blue Carbon habitats?
- can planning and management ameliorate the threat that climate change presents to Blue Carbon stores?
- do Blue Carbon habitats reduce the lability (and therefore increase storage) of terrestrial Corg from the watersheds?
- how can we include seagrass Blue Carbon into national inventories, given the need to redefine the land area of nations to include subtidal habitat?

John Raven

- What are the implications of changes in global cover of the coastal macrophytes on DMS and halocarbon release with direct and indirect effects on radiative forcing?
- Are there any long-lived biomarkers from coastal macrophytes (or other sources) in organic carbon deposits that can help with determining the source of the organic carbon and its radiocarbon age, using techniques pioneered by Tim Eglinton?

Karen McGlathery

- What are the sources of sediment organic carbon in seagrass meadows? How much is allochthonous vs. autochthonous? What is the important of in situ, non-seagrass productivity (i.e., benthic micro algae) to carbon storage in seagrass sediments? How do the source contributions vary spatially with meadow size/configuration and proximity to adjacent ecosystems (e.g., marshes)? How can this information inform policies on carbon accounting?

- How can restoration reinstate carbon storage/sequestration capacity? What is the time frame over which this occurs? Are the "best practices" that can be recommended for seagrass restoration projects that focus on carbon sequestration? Can we manage ecosystems to sequester more carbon?
- How does nutrient enrichment affect metabolism and carbon sequestration in seagrass biomass and sediments? How do the source contributions vary with nutrient enrichment?
- What is the resilience of buried carbon to climate change? For example, how does temperate effect carbon metabolism and storage, especially in the context of temperature-related seagrass die-offs?
- How important is carbonate chemistry to net CO2 fluxes in seagrass meadows in temperate and tropical regions?

Tomohiro Kuwae and Kenta Watanabe

- Tradeoffs (dilemma) between carbon storage and CO2 emission caused by carbonate production and organic matter decomposition in coastal BC ecosystems (e.g., Kuwae et al., 2016)
- Extension of BC studies to seaweed ecosystems (Krause-Jensen and Duarte, 2016) and coral reefs
- Synergies and tradeoffs between BC functioning (mitigation and adaptation) and other ecosystem services (fisheries, recreation, water quality improvement)
- Tradeoffs between CO2 emission (negative for mitigation) and land formation (positive for adaptation) by calcifiers

Mark Huxham

- What is the total quantity of carbon stored in the various key Blue Carbon sinks?
- What are the rates of loss and degradation of Blue Carbon habitats?
- How does loss and degradation affect carbon storage in Blue Carbon habitats?
- What are the rates of re-oxidation of carbon (or carbon loss) from Blue Carbon sinks following habitat loss or degradation?
- What are the key drivers of habitat loss and degradation in different areas of the world?

- How can we best communicate to the public and to policy makers the importance of Blue Carbon habitats? (this includes the secondary question of whether an emphasis on carbon implicitly devalues the other ecological services and the intrinsic beauty and value of these habitats).
- What management interventions have been shown to work and what are the local contexts in which they best succeed?
- What are our best predictions for the effects of climate change on carbon dynamics and persistence of these systems? For example, under what conditions may sinks become sources or sulphate reduction no longer limit methanogenesis?
- Does the use, promotion and expansion of payments for ecosystem services schemes (particularly carbon offsetting) involving Blue Carbon habitats lead to perverse outcomes such as 'moral hazard'; a failure to deal with the larger structural and behavioural changes need to tackle climate change?
- Can we develop relatively simple protocols, measurements and tools to allow non-specialists and local groups to assess and communicate the value of their Blue Carbon ecosystems and integrate these into international processes such as IPCC?

Nicola Beaumont

- How do we define permanence in carbon storage? For example do we consider permanent storage to be 100 years, or a millennia?
- How do we include risk in the estimates of permanence of storage? For example we may know a seagrass bed stores 100tonnes of carbon per year and this will be permanent if conditions remain the same. However, it is possible that changes may occur (port development, pollution, significant storms) which will remove or destroy the seagrass bed, possibly also releasing the carbon stored. Including this risk element in our estimates of carbon storage is critical to managers.
- How do we handle spatial boundaries in Blue Carbon science? I have seen many studies that report export of carbon from a given system with no consideration of what will happen next. We need more joined up thinking to understand how carbon moves between systems.
- How do we include uncertainty in our estimates of Blue Carbon sequestration and storage? These are critical to policy makers and managers but are rarely reported in a consistent or

clear fashion. I would advocate a move towards standardised approaches to documenting uncertainty.

- How do we monetarily value Blue Carbon in a meaningful way? There are a variety of monetary values which we can associate with Blue Carbon and this can be done relatively easily to provide a value, but little research has been undertaken to investigate the validity of these values. For example: which monetary value is most applicable to which environment? - the values vary by more than an order of magnitude, so the value you choose makes a significant difference to the final value. Which discount rate should be applied? There have been no detailed studies into methodological and conceptual development of valuing Blue Carbon and this is a real gap in the research.
- What options are there to value Blue Carbon beyond monetary estimates? There are a host of difficulties in monetising Blue Carbon (as above) so what other approaches can we take?

Nuria Marba

- global current extent of seagrass meadows, salt-marshes, macroalgae
- thickness of carbon stores that can act as C source in degraded seagrass meadows
- magnitude of carbon emissions from degraded seagrass meadows
- creation of new BC carbon sinks (e.g. macroalgae farming; seagrass carbon sinks in new suitable areas as e.g. sub-Arctic and Arctic)
- co-benefits of BC

Oscar Serrano

- Should allochthonous C be accounted for in BC accounting?
- How to estimate allochthonous C robustly in BC ecosystems?
- what's the change in Corg stock and acc rates after habitat loss?
- what's the fate of Corg stock loss after habitat loss?
- What's the role of BC ecosystems in climate change mitigation and adaptation over different time-scales? from present to geological scales.
- Area of BC ecosystems (particularly critical for seagrass and tidal salt marsh)?
- Produce a global, robust, standard dataset that could assist NGO, Industry etc to establish policy and crediting schemes

- Create BC data to fulfil gaps from underrepresented areas/regions
- Involve industry into BC initiatives (e.g. BC international workshop)
- Carbonate accounting in CO2 cycling and fluxes
- What is autochthonous C? Everything that happens in the area boundaries occupied by BC ecosystems
- Macroalgae and standing stocks in living BC biomass are BC sinks? Depends on the fate of biomass or the C footprint of e.g. Food and other bioproducts
- Biochar/fertilisers from wrack is a friendly solution for managing wrack waste and reduce emissions
- Estimate the export of BC biomass into other ecosystems (beach wrack, deep ocean, adjacent ecosystems, etc.)
- Evaluate cost/benefit (feasibility) of BC projects: need to incorporate the $value of additional ecosystems services (and create markets for them if don't exist).
- Role of BC ecosystems in keeping pace with SLR: need to estimate soil accretion but also their role in supporting calcifying organisms and export of biogenic sands

Patrick Megonigal

- How do biogeochemical, geomorphic and hydrologic factors interact to preserve carbon, and how does the relative importance of these factors vary spatially? The basic controls are well known, but there is increasing evidence that we do not understand the interactions well enough to develop spatial maps from databases and remote sensing products.
- How do biogeochemical, geomorphic and hydrologic factors interact to control the fate of carbon exported from intact or disturbed Blue Carbon ecosystems? Again, we understand the basic processes, but are far from having coupled wetland-estuarine models that can predict the outcome of an erosion event (for example).
- Considering the fact that carbon sequestration and methane emissions are biogeochemically linked processes, under what circumstances are climate benefits maximized when protecting, restoring and creating Blue Carbon ecosystems?

Paul Lavery

- Emissions factors for different ecosystems in both baseline and disturbed conditions. This needs to encompass N oxides and methane as well as CO2. It also needs to take into account the different biogeochemical settings and the the time-course of responses following restoration or management interventions aimed at reducing emissions.
- Carbonate production – exactly how important is this, in what ecosystems is it important and what is the geographical distribution of those ecosystems. How do we factor this into our estimates of net C accumulation in BC ecosystems. This needs serious attention from geochemists that that consider the simultaneous precipitation and dissolution processes occurring in ecosystems.
- Macroalgae – it is currently a theoretical contribution, and maybe a big one, but we need to get empirical evidence. We need a global network on this to establish how significant it is and which factors in the variability that may be associated with the distribution of major macroalgal production hotspots.
- Allochthonous: Autochthonous ratio – this is still relevant to Corg but is likely to be even more so for carbonate
- Extent of BC habitats – definitely not sexy, but it remains a critical knowledge gap. All our efforts to reduce the errors in stocks estimates by refining carbon density measures can be easily undone by the very poor estimates of BC habitat extent.
- Climate change impacts – in particular, there is uncertainty about how changes such as sea level rise will affect BC ecosystems. The effects may not be consistent across all situations so we need to get a better conceptual framework for assessing this. Another critical aspect is how tropicalisation, resulting from climate change, may affect BC ecosystems. The expected shifts in BC primary producers and their grazers could have complex, interactive effects on BC stocks and accumulation rates.
- Filling in the geographical gaps in stock – Coral triangle is woefully under-represented in our global syntheses and saltmarshes are poorly captured compared to the other ecosystems.
- What are the impediments to uptake/incorporation of BC into carbon crediting schemes and how can these be overcome. This is more of an economic/socio-ecological question, but one which needs to be addressed if we are to get traction in the crediting community.
- How can we value the carbon sequestration service of BC ecosystems? Currently we rely on fairly simple estimates based on anticipated C-trading scheme values. but in the same way

that fisheries values are much more extensive than the direct sale price of the fish, are there other aspects of valuation that we need to take into account?

Pere Masque

- Relevance of CO2 released by carbonate sediment production
- Adequate assessment of net Corg burial rates at various time scales: year, decades and centuries
- Assessment of remineralisation rates of Corg in the soils attending to its various types
- Assessment of spatial coverage of vegetated coastal habitats at regional and global scales, particularly for seagrass meadows
- Fate of Corg after disturbance of vegetated sites: remineralisation vs redistribution
- Assessment of macroalgae in carbon sequestration: where does the Corg go? (i.e. build on Krause-Jensen and Duarte)

Rod Connolly (with comments on the table)

- What generalised models best predict spatial variation in rates of BC production?
- How can the fate of C produced in wetlands be more rigorously allocated to BC vs other routes (e.g. grazing, decomposition, export)?
- How does seascape influence BC production?
- How does BC valuation for CC mitigation compare with valuation of labile C supporting seafood production?
- How will current and future climate feedbacks affect BC production?
- How do different disturbances, from temporary shallow to permanent deep, affect the amount of existing and future BC production?
- What is the impact of ocean sprawl on BC production?
- How can urbanised and industrialised wetlands be managed (modified) to maximise BC production?
- Is widespread eutrophication of coastal waters stimulating or stymying BC production?
- How can frequency and extent of inundation of mangroves be managed to optimise BC production?

Rui Santos

- What is the proportion of autochthonous versus allochthonous carbon that is sequestered into the sediments of mangrove, tidal marsh and seagrass ecosystems?
- What is the role of water flow (and turbulence) on allochthonous blue C sequestration (as mediated by the sediment grain size)?
- What is the natural turnover time of Blue Carbon sequestered in the sediments of mangrove, tidal marsh and seagrass ecosystems?
- How do sediment properties and microbiota affect this turnover?
- How do anthropogenic disturbances affect the release of Blue Carbon back to the atmosphere?
- What is the proportion of organic carbon exported from mangrove, tidal marsh, macroalgal, and seagrass ecosystems that is sequestered?
- How much carbon is sequestered by fleshy macroalgal beds (and marine algal crops)?
- Are rhodolith beds sources or sinks of carbon? Will OA alter their role by increasing dissolution versus precipitation?
- How does calcification offsets the C sequestration by seagrasses? How will OA and temperature affect this balance?
- Will Blue Carbon sequestration increase in a high CO2 future?
- What is the C sequestration potential of reconstructed ecosystems? How much time do they need to equal natural ecosystems?
- How relevant is Blue Carbon sequestration of reconstructed ecosystems for climate change mitigation?
- How relevant is Blue Carbon sequestration versus other ecosystem services provided by mangrove, tidal marsh, macroalgal, and seagrass ecosystems?

Thomas Bianchi

- How deep to we really need to core to get the best long-term rates of sequestration and how do they differ across BC habitats?
- How important is it to determine other sources of carbon (e.g., algal or seagrass) when estimating the carbon stores of a particular habitat (e.g. mangroves)?

- How the rates of decay vary with depth and what are the controlling mechanisms across different BC habitats and regions?
- What are the GHG emissions from these systems?
- How important is later import and export of allochthonous OC material from and to these systems?
- What is the impact of relative sea-level rise and global warming on C sequestration rates in BC habitats, and can they be separated?
- What is the fate of eroding BC in coastal systems?
- Can we establish a universal worldwide system for carbon trade on preservation of these systems?
- How is global warming change the composition of coastal BC habitats (marsh to mangrove) and what are the benefits or losses from this transition.
- How can coastal plans for river diversion in regions experiencing high land loss (e.g., Mississippi, Shanghai, etc.) be combined with the added value of wetland services performed BC C sequestration to enhance the efficacy of coastal planning and management.

Tiziana Luisetti

- What are the functioning requirements of coastal Blue Carbon (e.g. mangrove, tidal marsh, macroalgal, and seagrass ecosystems) to be economically valued?
- What is the cost to society of losing Blue Carbon, or the gain for restoring it?
- How much carbon is released back into the atmosphere following anthropogenic disturbance on coastal 'blue' carbon?
- What is the cost to society of re-emitted carbon from coastal Blue Carbon stocks/sinks?
- What are the bio-physical and economic requirements needed to include coastal Blue Carbon in a global carbon permit trading market?
- What international agreements are needed to allow coastal Blue Carbon permits to be traded?
- What policies are needed to protect coastal Blue Carbon?

Trisha Atwood

- What role do macroalgal systems play in long-term carbon storage?

- How, and to what extent, do above-ground processes like herbivory influence carbon accumulation and retention in Blue Carbon and macroalgal ecosystems?
- To what degree does adjacent land use influence sources of carbon and sedimentation rates to these systems?
- How do above- and below-ground plant traits influence carbon accumulation and retention?
- Can we make generalizations about the fate (transported or transformed) of disturbed soil C
    - How does disturbance type influence the fate of disturbed soil C
    - What is the magnitude of loss (transformed or transported) and how deep in the soil matrix does that loss occur.
